# Characterization of diazotrophic root endophytes in Chinese silvergrass (*Miscanthus sinensis*)

**DOI:** 10.1186/s40168-022-01379-9

**Published:** 2022-11-03

**Authors:** Yongbin Li, Rui Yang, Max M. Häggblom, Mengyan Li, Lifang Guo, Baoqin Li, Max Kolton, Zhiguo Cao, Mohsen Solemani, Zheng Chen, Zhimin Xu, Wenlong Gao, Bei Yan, Weimin Sun

**Affiliations:** 1 Guangdong Key Laboratory of Integrated Agro-Environmental Pollution Control and Management, Institute of Eco-Environmental and Soil Sciences, National-Regional Joint Engineering Research Center for Soil Pollution Control and Remediation in South China, Guangdong Academy of Sciences, Guangzhou, 510650 China; 2 Joint Laboratory for Environmental Pollution and Control, Guangdong-Hong Kong-Macao, Guangzhou Institute of Geochemistry, Chinese Academy of Sciences, Guangzhou, 510640 China; 3 Department of Biochemistry and Microbiology, Rutgers University, New Brunswick, NJ 08901 USA; 4 Department of Chemistry and Environmental Science, New Jersey Institute of Technology, Newark, NJ 07102 USA; 5 French Associates Institute for Agriculture and Biotechnology of Drylands, Ben-Gurion University of the Negev, Beer Sheva, Israel; 6 School of Environment, Key Laboratory of Yellow River and Huai River Water Environment and Pollution Control, Ministry of Education, Henan Normal University, Xinxiang, 453007 China; 7 Department of Natural Resources, Isfahan University of Technology, Isfahan, Iran; 8 Department of Health and Environmental Sciences, Xi’an Jiaotong-Liverpool University, Suzhou, 215123 China; 9 Engineering and Technology Research Center for Agricultural Land Pollution Prevention and Control of Guangdong Higher Education Institutes, College of Resources and Environment, Zhongkai University of Agriculture and Engineering, Guangzhou, 510225 China

**Keywords:** Diazotrophic endophytes, Stable isotope probing (SIP), Metagenomic-binning, Bacterial colonization

## Abstract

**Background:**

Phytoremediation is a potentially cost-effective way to remediate highly contaminated mine tailing sites. However, nutrient limitations, especially the deficiency of nitrogen (N), can hinder the growth of plants and impair the phytoremediation of mine tailings. Nevertheless, pioneer plants can successfully colonize mine tailings and exhibit potential for tailing phytoremediation. Diazotrophs, especially diazotrophic endophytes, can promote the growth of their host plants. This was tested in a mine-tailing habitat by a combination of field sampling, DNA-stable isotope probing (SIP) analysis, and pot experiments.

**Results:**

Bacteria belonging to the genera *Herbaspirillum*, *Rhizobium*, *Devosia*, *Pseudomonas*, *Microbacterium*, and *Delftia* are crucial endophytes for Chinese silvergrass (*Miscanthus sinensis*) grown in the tailing, the model pioneer plant selected in this study. Further, DNA-SIP using ^15^N_2_ identified *Pseudomonas*, *Rhizobium*, and *Exiguobacterium* as putative diazotrophic endophytes of *M. sinensis*. Metagenomic-binning suggested that these bacteria contained essential genes for nitrogen fixation and plant growth promotion. Finally, two diazotrophic endophytes *Rhizobium* sp. G-14 and *Pseudomonas* sp. Y-5 were isolated from *M. sinensis*. Inoculation of another pioneer plant in mine tailings, *Bidens pilosa*, with diazotrophic endophytes resulted in successful plant colonization, significantly increased nitrogen fixation activity, and promotion of plant growth*.*

**Conclusions:**

This study indicated that diazotrophic endophytes have the potential to promote the growth of pioneer plant *B. pilosa* in mine tailings.

Video Abstract

**Supplementary Information:**

The online version contains supplementary material available at 10.1186/s40168-022-01379-9.

## Background

Mine tailings are fine-grained minerals (1–600 μm) generated from the processing of ores [1]. It is estimated that more than 10 billion tons of waste are produced from mine activities annually [2]. The contamination introduced by mine tailings represents a global environmental challenge because they can leach toxic metal(loid)s, such as antimony (Sb) and arsenic (As) [3–5]. Mine tailings heavily contaminate China, with over 10 billion tons of tailings produced since 2009 [6]. Phytoremediation using living plants to clean up contaminated soils has many potential benefits over traditional physical and chemical cleanup approaches. It is minimally disruptive, cost-efficient, and has high levels of public acceptance [7, 8]. For these reasons, phytoremediation is well suited to manage and remediate mine tailing contamination [9]. Re-vegetation is the first step for tailing phytoremediation and offers a promising way to reduce the environmental impact of tailings. Pioneer plants that can tolerate high metal(loid) concentrations are ideal for tailing re-vegetation and support the further establishment of other plant species by increasing soil nutrients [10] and/or reducing soil acidity [10, 11]. Since oligotrophic (e.g., C, N, P deficiency) mine tailings [12–14] are unfavorable environments for plant growth, it is important to understand how the pioneer plants gain sufficient nutrients in mining tailings and whether similar mechanisms can be applied for other plants.

Nitrogen (N) is an essential factor that regulates the growth of microorganisms and plants, governing the efficiency of phytoremediation of mine tailing [15–17]. Biological N fixation (BNF) mediated by diazotrophs has important environmental implications in tailings by supplementing N to plants and may contribute to phytoremediation [18]. Previous studies observed the enrichment of diazotrophs and their important environmental roles in tailings relatively to adjacent soils [19, 20]. Unlike rhizosphere-associated diazotrophs, diazotrophic endophytes live within plant tissues and establish themselves in less competitive niches with more favorable conditions for N fixation [21–23]. Many diazotrophic endophytes hold the potential to promote plant growth in monocot crops (e.g., sugarcane, rice, and maize), dicot crops (e.g., sweet potato and coffee), and bioenergy crops (e.g., poplar and willow) [24–30]. For example, sugarcane inoculation with diazotrophic endophytes resulted in a 40% higher fresh cane weight than those inoculated with a dysfunctional mutant for N_2_ fixation [31]. Additionally, inoculating ryegrass plants with diazotrophic endophytes, isolated from native poplar grown under nutrient-poor conditions, significantly increased plant biomass [32]. Thus, endophytic diazotrophic bacteria can potentially promote the establishment of pioneer plants in tailings and thus contribute to tailing vegetation and phytoremediation.

*Miscanthus sinensis* (Chinese silver grass) is a pioneer perennial grass plant native to eastern Asia and extensively encountered in mine tailings [33]. *Miscanthus* is known for its high metal accumulation potential and biomass production, making it well suited for phytoremediation of mine tailings [34]. Moreover, *M. sinensis* root-associated microbiome may contribute to metal resistance, nutrient acquisition, and promote plant growth [20]. Hence, *M. sinensis* was selected as the model pioneer plant for this study. We sampled *M. sinensis* from 4 mine tailings across Southwest China. Initially, an endospheric microbiome of *M. sinensis* was characterized and compared with those grown at less contaminated sites. Then, DNA stable isotope probing (DNA-SIP) using ^15^N_2_ coupled with metagenomic-binning analysis was performed to identify putative diazotrophic endophytes and predict their metabolic potentials. This approach links microbial identity with environmental function and provides a better understanding of active diazotrophic communities without isolation and cultivation. Finally, to assess the capability of diazotrophic endophytes to promote plant growth, *Bidens pilosa*, another plant frequently detected in mine tailings with rapid growth capability, was inoculated with native diazotrophic endophytes isolated from *M. sinensis* (see Figure S1 for overview of the experimental design). The current study aimed to (i) investigate the endospheric keystone taxa of *M. sinensis* that grow in mine tailings; (ii) identify diazotrophic endophytes of *M. sinensis*; and (iii) assess the feasibility of inoculated diazotrophic endophytes to promote plant growth in mine tailings.

## Methods

### Sample collection and preparation

A total of 20 M*. sinensis* root samples were collected from 4 mine tailings across Southwest China (5 root samples per sampling site). Control root samples were collected from nearby less contaminated sites (see Figure S2 for details). Chemical properties of sampling sites were measured and summarized in Table S1. Plant root samples were collected using an ethanol sterilized shovel and kept on ice in sterile bags.

DNA from root endosphere was extracted as described previously [20]. Roots were washed with TE-buffer (adding 2% Tween 20, pH 7.5), surface-sterilized using 2% NaClO for 10 min, and then washed using 70% ethanol 5 times. Finally, the root samples were washed with sterile deionized water for 4 times. The last wash was spread on LB gar plates to check whether microorganisms were removed [35]. Then, root samples were ground with liquid N, and DNA was extracted with a DNeasy Powersoil kit [36] according to the manufacturer's protocol (QIAGEN, Dresden, Germany). The quality and quantity of DNA were examined by the NanoDrop 2000 spectrophotometer (Thermo Fisher Scientific, Waltham, MA, USA).

### Identification of diazotrophic endophytes using DNA-SIP

#### Extraction of root endophytic microorganisms

Root samples of *M. sinensis* taken from the Xikuangshan (XKS) site were chosen to extract the cultivable endophytic microorganisms using a Nycodenz density-gradient centrifugation method [37–39]. To ensure only endophytes were extracted, the surface sterilization was further confirmed by applying wash buffer (R1, R2, R3) to LB agar plates. For the wash buffer R3, no growth was observed after 7 days of incubation at 28 °C (see Figure S3 for details), suggesting that no root-surface-associated live microorganisms were isolated. Therefore, bacteria identified by DNA-SIP or used in pot experiments were presumably root endophytes because only cultivable microorganisms were targeted in these two experiments.

##### DNA-SIP

DNA-SIP was conducted to demonstrate nitrogen fixation using ^15^ N-labelled versus ^14^ N-labelled N_2_. Endospheric bacterial cultures mentioned above was used as inoculants to set up cultures for DNA-SIP. First, 20% N-free Jensen's broth (broth:distilled water = 1:5 (*v:v*)) (M973, HiMedia Laboratories, Mumbai, India) was replaced with 30 mL mixed gas (^15^N_2_(or ^14^N_2_):O_2_ = 8:2 (*v:v*)) in a 60-mL serum bottle using the drainage method. Then, approximately 1 mL of the extracted microbial culture was mixed with the remaining 30 mL broth using a sterile syringe. All microcosms were incubated at 30 °C at 180 rpm in the dark and were destructively sampled on days 21, 28, and 35.

#### SIP gradient fractionation

The extracted DNA of SIP incubations was separated into “heavy” (i.e., ^15^ N-DNA) and “light” (i.e., ^14^ N-DNA) fractions by CsCl gradient ultracentrifugation (Additional files, [13]).

### Sequencing of 16S rRNA

DNA from *M. sinensis* roots collected from tailings and DNA-SIP treatments ^14^N_2_ and ^15^N_2_ (triplicate samples, day 28 and day 35) were used for amplicon sequencing of the V4 of 16S rRNA gene using 515F/806R [40]. The products were sequenced at Personal Biotechnology (Shanghai, China). The raw reads were trimmed and quality controlled by QIIME2, and chimeras were removed by DADA2 [41]. Representative amplicon sequence variants (ASVs) were assigned against the SILVA 132 database. The alpha diversity, PCoA, and co-occurrence network were analyzed based on the previous description [42].

### Shotgun metagenomic sequencing

Since the mass of DNA in a single heavy fraction of the SIP samples was insufficient for sequencing, the heavy fractions with *nifH* copy numbers from each of the triplicate ^15^ N treatments were pooled as one composite DNA sample, which was sequenced at Personal Biotechnology (Shanghai, China) (Additional files).

### Diazotroph isolation and phylogenetic analysis

Cultures from the DNA-SIP assay at day 35 were used for the isolation of the diazotrophic bacteria. Briefly, the cultures were serially diluted in 0.9% NaCl solution (up to 10^−5^) and then screened on agar plates containing the N-free Jensen's medium (M710). The plates were incubated at 30 °C for 3–5 days, and colonies were further purified and characterized. Then, PCR amplification of the *nifH* gene was performed to screen for diazotrophs [43]. An acetylene reduction assay was conducted to measure the nitrogenase activity to confirm the capability for nitrogen fixation [43]. The near full-length 16S rRNA gene was amplified with the primer pair 27F/1492R and sequenced, and sequences were annotated against the NCBI database. The neighbor-joining phylogenetic tree was constructed with MEGA. The morphology of the isolated strains was examined by scanning electron microscopy (Phenom proX, Phenom-World BV, Netherlands).

### Growth promotion potential of isolated diazotrophs

A pot experiment was employed to assess the growth promotion potential and colonization ability of the isolated diazotrophs on *B*. *pilosa* L. Four different treatments in triplicate were established as follows: (i) inoculation with *Rhizobium* sp. G-14 (designated as Rhi.Inoc.), (ii) inoculation with *Pseudomonas* sp. Y-5 (designated as Pseu. Inoc.), (iii) inoculation with the mixture of *Rhizobium* sp. G-14 and *Pseudomonas* sp. Y-5 (designated as Mix. Inoc.), and (iv) treatments without inoculating pure isolates (designated as control) (Additional files).

### ^*15*^*N*_*2*_* enrichment incubation assay*

To determine whether the diazotrophic endophytes facilitate N fixation, a ^15^N_2_ enrichment incubation assay was conducted by planting *B. pilosa* L. in a sterile sealed anaerobic tube (please see Figure S4 for details of the experimental setup). The mixture of tailings and vermiculite (*v:v* = 1:1) was added to the 50 mL sterile serum tubes. Seeds of *B. pilosa* L. and 3 mL bacterial suspension obtained as described in the section of growth promotion potential of isolated diazotroph (or deionized water in control treatment) were added into the anaerobic tubes. After germination, the anaerobic tubes were sealed, and 10% (v/v) of the headspace was replaced with ^15^N_2_. After culturing for 2 days, the anaerobic tubes were ventilated again for 1 day, which is designated as one cycle. The samples, including rhizosphere soil, root, and shoot were collected destructively after 3 cycles and were freeze-dried for further ^15^ N isotope analysis. The δ^15^N value of processed plant tissue was determined by an isotope ratio mass spectrometer (IR-MS, DELTA V Advantage Thermo Fisher Scientific, Inc., Waltham, MA, USA).

### Statistical analysis and data availability

Student’s *t* test was performed to examine the significance of the differences in the microbial abundance and *nifH* relative abundance (*nifH* abundance/16S rRNA abundance) of *M. sinensis* root endosphere using the SPSS software v.20. A one-way analysis of variance (ANOVA) with the least significant difference (LSD) test was conducted to determine the significance of the differences in fresh weight, ^15^ N abundance, N content, the relative expression of *nifH*, and *Rhizobium*/*Pseudomonas* abundances of *B*. *pilosa* using SPSS v20.0. The raw data have been submitted to the NCBI database for public use (accession no PRJNA818089).

## Results

### Bacterial community and keystone taxon analysis of the M. sinensis root endosphere

**Fig. 1:**
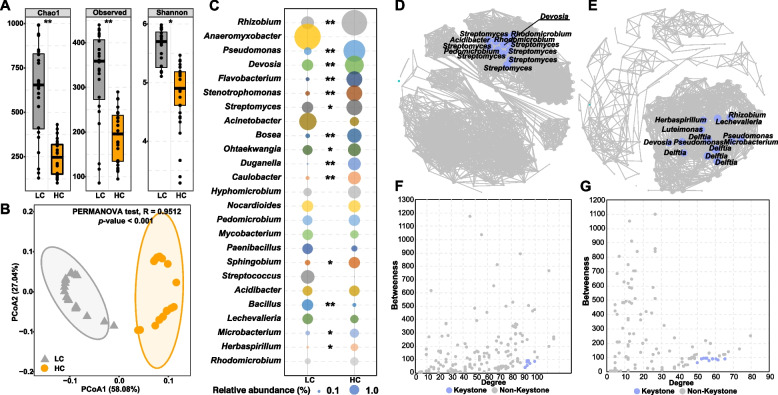
The boxplots show the bacterial alpha diversity indices in the root endosphere at the low contaminated (LC) and the high contaminated (HC) sites (**A**). The PCoA plot of beta similarities was measured as Bray–Curtis distances for the bacterial community (**B**). Comparison of the different genera distribution between LC and HC sites (**C**). * and ** indicate significant differences between the LC and HC at *p* < 0.05 and *p* < 0.01, respectively. Co-occurrence network analysis showing the biological interactions in the LC (**D**) and HC (**E**). Edges are shown only strong (Spearman correlation >|0.6|) and significant (*p* < 0.05) connections. The size of the nodes is proportional to the number of connections to it. The scatter plot shows criteria of selecting for the keystone taxa in the LC (**F**) and HC (**G**). The bacterial community was analyzed based on 5 replicate root samples from each sampling site (a total of 20 root samples)

*M. sinensis* plants were collected from four highly contaminated tailing sites (HC) and from adjacent sites with lower contaminant levels (LC). The endospheric microbial communities were extracted and characterized. Microbial diversity analysis indicated that lower alpha diversities (e.g., Chao1, Observed, and Shannon) of the endosphere microbiome were observed in the HC samples compared to those of LC (Fig. 1A). Bacterial community compositions were significantly different between the HC and LC based on the Bray–Curtis similarity (PERMANOVA, *R* = 0.95, *p* < 0.01) as visualized by two distinct clusters (Fig. 1B). The microbial community compositions indicated different distributions of the dominant microbial community members. *Proteobacteria*, *Actinobacteria*, and *Bacteroidetes* ranked as the top 3 most abundant phyla (see Figure S5 for details). *t* test analyses further demonstrated that *Proteobacteria* was dominant in HC while *Planctomycetes*, *Acidobacteria*, and *Firmicutes* were dominant in LC. Further analysis at the genus level indicated that while *Rhizobium* was the most dominant genus in the HC, accounting for 5.5% of the relative abundance (Fig. 1C), *Anaeromyxobacter* was the most dominant genus in the LC, accounting for 5.7% of the relative abundance. In addition, while the genera *Rhizobium*, *Pseudomonas*, *Devosia*, *Flavobacterium*, and *Stenotrophomonas* were significantly enriched at HC sites, members of *Bacillus* were abundant at LC sites (Fig. 1C). Further, keystone taxa were identified according to the criteria of the nodes with low betweenness centralities and high degree as reported previously [44, 45] (Fig. 1F, G). In LC, 12 ASVs were identified as the keystone taxa (see Fig. 1D and Tables S2 and S3 for details). In contrast, the keystone taxa in the HC network were significantly different (see Fig. 1E and Tables S4 and S5 for details). Notably, the identified keystone taxa were different in LC and HC sites except *Devosia*. In addition, the high *nifH* relative abundance (*nifH* abundance/16S rRNA abundance, Figure S6) were observed in the HC sites.

### Diazotrophic endophytes identified by DNA-SIP

**Fig. 2:**
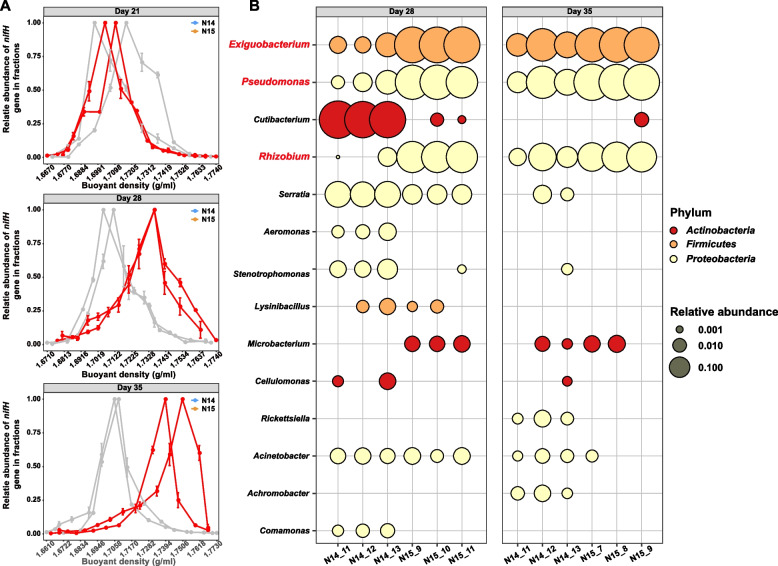
**A** Relative abundances of *nifH* from the treatment ^14^ N and ^15^ N after incubation. The abundance of the *nifH* gene in each fraction was converted to the proportion of total *nifH* gene abundance throughout the gradient fractions. **B** Relative abundances of 14 most abundant microbial genera in light, ^14^ N treatment, and heavy ^15^ N DNA fractions at days 28 and 35. Each bubble stands for one DNA fraction. Triplicate fractions were sequenced for each treatment

DNA-SIP was conducted to identify *M. sinensis* root endophytes with diazotrophic capacities. Accordingly, ^15^N_2_ was applied to cultures inoculated from root endosphere bacterial extracts. The relative abundances of *nifH* across each fraction at three time points (e.g., days 21, 28, and 35) are shown in Fig. 2A. The gradual incorporation of ^15^N_2_ into the DNA of endophytes was observed as a proxy for N fixation. The maximum relative abundances were initially detected in the light fractions (with buoyant density (BD) values of 1.71 g mL^−1^ in the ^14^N_2_ treatment on day 21) and gradually shifted to the heavier fractions in the ^15^N_2_ treatment.

The fractions with the highest relative abundances of *nifH* genes on days 28 and 35 were selected for amplicon-based metagenomic analysis of the 16S rRNA gene (Fig. 2B). Bacteria taxonomically affiliated with *Exiguobacterium*, *Pseudomonas*, and *Rhizobium* dominated in the heavy DNA fractions of the ^15^N_2_-labeled treatments while not in the corresponding light fractions, suggesting the potential role of *Exiguobacterium*, *Pseudomonas*, and *Rhizobium* as diazotrophic endophytes for *M. sinensis*.

### Metabolic potentials of putative diazotrophic endophytes

**Fig. 3:**
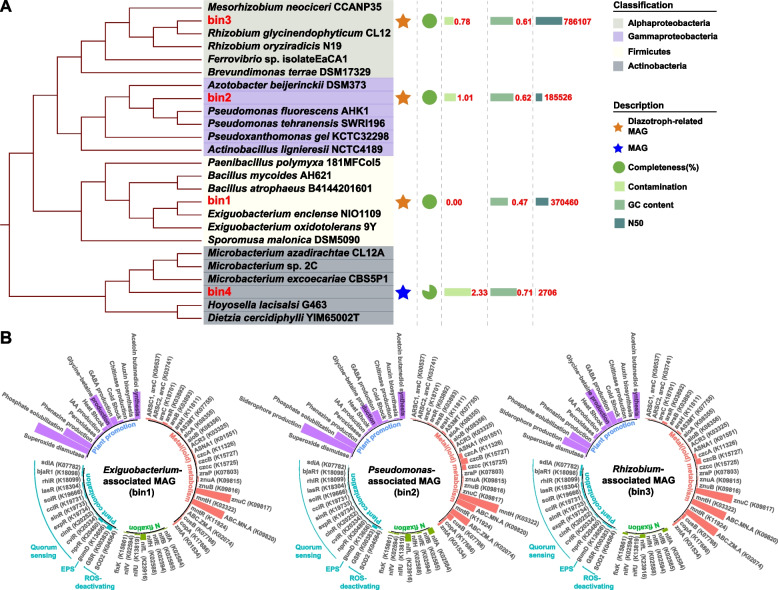
Phylogenomic tree of MAGs from the metagenome of heavy DNA fractions. The genes encoding nitrogen fixation, plant colonization, plant growth promotion, and metal(oid) resistance in the MAGs associated with putative diazotrophs (**B**). The height of the bar indicates gene abundance

Four high-quality metagenome-assembled genomes (MAGs) with > 75% completeness and < 5% contamination were phylogenetically classified to the genera *Exiguobacterium*, *Pseudomonas*, *Rhizobium*, and *Microbacterium* (Fig. 3A). MAGs associated with putative diazotrophic endophytes *Exiguobacterium*, *Pseudomonas*, *Rhizobium* identified by DNA-SIP were designated as bins 1, 2, and 3, respectively. Genes related to N_2_ fixation were identified in all these three MAGs (Fig. 3B). In addition, genes related to plant colonization such as quorum sensing system, ROS-deactivation, and EPS production were detected in all three MAGs (Please see “Discussion” section for details) (Fig. 3B). These MAGs also contain genes for metal(loid)-resistance (e.g., arsenic, nickel, cobalt, zinc, manganese, copper, and cadmium) and plant growth promotion (PGP) (e.g., phosphate solubilization function, siderophore production, gamma-aminobutyric acid production, and acetoin/butanediol synthesis).

### Isolation of diazotrophic endophytes and their capability for plant growth promotion

Two diazotrophic endophytes, *Pseudomonas* sp. Y-5 and *Rhizobium* sp. G-14 were isolated from the root endosphere of *M. sinensi*s (see Figure S7A, B for details). Notably, both isolates are phylogenetically relevant to the putative diazotrophic endophytes identified by DNA-SIP. Consequently, acetylene reduction analyses of nitrogenase activity confirmed BNF capability of the isolates (Figure S7C). Unfortunately, no diazotrophic endophytes belonging to *Exiguobacterium* were recovered through conventional plating and isolation.

**Fig. 4:**
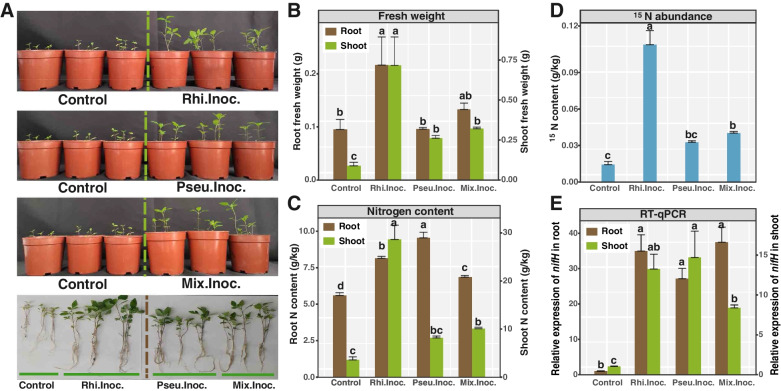
**A** Pictures of seedlings of *Bidens pilosa* L. with different treatments. **B** Effects of isolated strain inoculation on fresh weight, **C** nitrogen content, and **D** ^15^ N abundance. Comparison of the *nifH* transcript level of the diazotrophs among the different treatments in root and shoot **E**. Values are given as a mean of 3 independent biological replicates, and the bars represent standard error. ANOVA with an LSD test (*p* < 0.05) indicated statistically significant differences denoted by different letters for each assessed parameter

Pot experiments were conducted to assess the capability of these bacteria to promote the growth of another tailing pioneer plant, *B. pilosa* L. (Fig. 4A). Three treatments with inoculated bacteria (i.e., Rhi.Inoc., Pseu.Inoc., and Mix.Inoc) significantly increased the shoot fresh weight of *B. pilosa* compared to the unamended control (Fig. 4B). In addition, inoculation of *P.* Y-5, *R.* G-14, or both cultures resulted in a significant increase of root/shoot N contents (Fig. 4C). Finally, ^15^N_2_ enrichment incubation and RT-qPCR of the transcribed *nifH* gene in shoots and roots of *B. pilosa* provided direct evidence demonstrating the promotion of N fixation by amending the diazotrophic endophytes: higher δ^15^N values were observed in the three inoculated treatments than those that did not receive amendments (Fig. 4D). Furthermore, RT-qPCR confirmed the expression of the bacterial nitrogenases in the roots and shoots of *B. pilosa*, since the copies of the transcribed *nifH* gene increased significantly in the shoots and roots of *B. pilosa* obtained from the inoculant treatments (i.e., Rhi.Inoc., Pseu.Inoc., and Mix.Inoc.) compared to their counterparts in the control treatment (Fig. 4E).

**Fig. 5:**
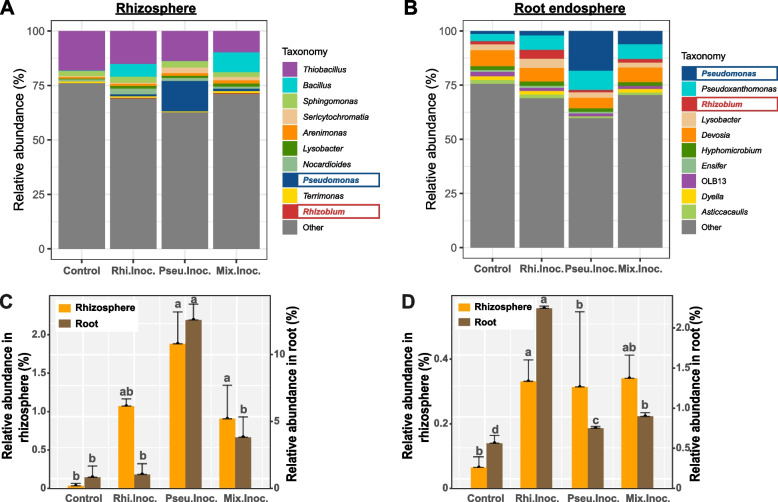
Composition of the bacterial community in the **A** rhizosphere and **B** root endosphere at the genus level. Arrows highlight the *Rhizobium* and *Pseudomonas*. Comparison of the distribution of **C** *Pseudomonas* and **D** *Rhizobium* among the different treatments. Values are given as a mean of 3 independent biological replicates, and the bars represent standard error. ANOVA with an LSD test (*p* < 0.05) indicated statistically significant differences denoted by different letters for each assessed parameter

Microbial community characterization of rhizosphere and root endosphere of *B. pilosa* L. was performed to verify the colonization of the amended bacteria. Based on the PERMANOVA analysis, inoculation treatments affected the bacterial community compositions in the rhizosphere (PERMANOVA, *R* = 0.388, *p* < 0.01, Figure S8A) and root endosphere (PERMANOVA, *R* = 0.69, *p* < 0.01, Figure S8B). Notably, the inoculation of amended bacteria significantly increased their abundance in the rhizosphere and root endosphere. For example, the relative abundances of *Pseudomonas* reached ~ 1.9% and ~ 12.6% in the rhizosphere and root endosphere of Pse.Inoc., respectively, while the relative abundances were only ~ 0.04% and ~ 0.84% in their control counterparts, respectively (Fig. 5A–C). The relative abundances of *Rhizobium* reached ~ 0.35% and ~ 2.25% in the rhizosphere and root endosphere of Rhi.Inoc., respectively, while the relative abundances were only ~ 0.07% and ~ 0.57% in their control counterparts, respectively (Fig. 5A, B, D). In addition, Mix.Inoc. significantly increased *Rhizobium* and *Pseudomonas* abundances in the rhizosphere and root endosphere. The increase of these amended bacteria in the two root-associated compartments, especially in the root endosphere, provided evidence of bacterial colonization. qPCR assay further confirmed that three treatments (Pse.Inoc., Rhi.Inoc., and Mix.Inoc.) significantly increased the abundances of *Pseudomonas* (Figure S9A, B, C) and *Rhizobium* (Figure S9D, E, F) in the rhizosphere and root/shoot endosphere compared to their control counterparts, supporting the colonization of these amended bacteria.

## Discussion

Vegetation on tailings is a promising bioremediation strategy to attenuate the contamination introduced by mine tailings. However, nitrogen (N) is one of the major limiting nutrients inhibiting plant growth in tailings [45]. Diazotrophic endophytes have the potential to provide the fixed N to improve the growth of host plants and promote the growth of pioneer plants in tailings and thus facilitate tailing vegetation. To verify such this hypothesis, putative diazotrophic endophytes in *M. sinensis*, a pioneer plant commonly detected in mine tailings, were identified and isolated. Further, their effects to promote the plants in mine tailings were examined.

### Putative diazotrophic endophytes as the keystone taxa of M. sinensis

*M. sinensis* is a pioneering plant that can thrive in metal-contaminated sites and holds the potential for phytoremediation applications [34, 46]. It is suggested that the endosphere microbiome of *M. sinensis* can provide critical environmental services, such as metal resistance and plant growth promotion, to support the survival of the host plant in mine tailings [20, 47]. This study characterized the endospheric microbiome of *M. sinensis* from four mine tailings (HC) and less contaminated (LC) sites. Particularly, the keystone taxa of *M. sinensis* were identified. The host-associated keystone taxa were referred to native microbial populations that were essential for the host biological functions [48]. Keystone taxa have been widely used to decipher the host-microbe interactions in different host-microbe ecosystems such as the mammalian guts or the plant roots [49, 50]. Identification of the keystone taxa of the meta–organism (i.e., *M. sinensis*) enabled the prediction of metabolic functions and core pathways provided by the host-microbe interaction [51]. Different bacterial taxa were dominant in the HC and LC soils. *Herbaspirillum*, *Lechevalieria*, *Rhizobium*, *Luteimonas*, *Devosia*, *Pseudomonas*, *Microbacterium*, and *Delftia* were dominant in highly contaminated tailing samples. Notably, most of these have previously been reported as diazotrophic bacteria [52, 53]. Consistently, significantly higher relative abundances of the *nifH* gene were observed in the root endosphere of HC samples than those of LC samples (Figure S6). These observations suggest the critical role of diazotrophic bacteria in the endosphere of *M. sinensis*.

### Putative diazotrophic endophytes identified by DNA-SIP

Subsequently, DNA-SIP was conducted to identify diazotrophic endophytes of *M. sinensis*. Accordingly, three bacteria affiliated with *Exiguobacterium*, *Pseudomonas*, and *Rhizobium* were identified as putative diazotrophic endophytes. Notably, two of these putative diazotrophic endophytes (i.e., *Pseudomonas* and *Rhizobium*) were identified as the keystone taxa in HC, indicating that N fixation may be an important metabolic trait of the endospheric microbiome of *M. sinensis*. Further, metagenomic-binning indicated that all these bacteria taxa contained the essential *nif* gene cluster for N fixation. In addition, MAGs associated with these three bacteria contained genes for plant growth promotion such as phosphate solubilization function, siderophores and gamma-aminobutyric acid (GABA) production, acetoin/butanediol synthesis, and metal resistance. These three bacteria taxa have previously been demonstrated with the capability for N fixation and plant growth promotion. For example, members of *Exiguobacterium* have been identified as nodule-associated bacteria from the root nodules of Fenugreek plant and showed their potential for N fixation [54]. *Pseudomonas lurida* EOO26, isolated from *Odontarrhena obovate* grown in the Cu contaminated soil, presented drought resistance, multi-metal tolerance, and exhibited PGP attributes including siderophore, 1-aminocyclopropane-1-carboxylic acid (ACC-) deaminase, and ammonia production [55]. In addition, as a potential plant for phytoremediation, *M. sinensis* might cause potential loss of soil N and P in its early and middle growth stages [34]. In this study, the identified diazotrophic endophytes also had P-solubilizing potential, further demonstrating the key role of diazotrophic endophytes in the nutrient uptake of *M. sinensis.*

### Plant growth promotion by diazotrophic endophytes

Previous studies suggested that diazotrophic endophytes can promote the growth of plants and thus increase crop yields [56]. Therefore, it is suggested that the diazotrophic endophytes of *M. sinensis* may also promote the growth of host plants inhabiting mine tailings. Accordingly, bacteria were isolated from the root extracts of *M. sinensis* to verify whether the endospheric bacteria can facilitate the BNF and promote plant growth. Two isolates (i.e., *Pseudomonas* sp. Y-5 and *Rhizobium* sp. G-14) closely related to the putative diazotrophic endophytes identified by DNA-SIP were obtained, and their capability to promote plant growth was tested and verified subsequently. Another pioneer plant, *B. pilosa*., was selected as a model plant in this study because it was frequently detected in mine tailings with the capability for rapid growth [57]. Pure isolates of *Pseudomonas* and *Rhizobium* were inoculated to the sterile mine tailing soils in which *B. pilosa* was planted. The addition of *Pseudomonas* and *Rhizobium* significantly improved the growth of *B. pilosa* (Fig. 4). All measured parameters, including length, fresh weight, and N content,were significantly higher in plants amended by *Pseudomonas, Rhizobium*, and the mixture of *Pseudomonas* and *Rhizobium* than their control counterparts. Moreover, significantly higher ^15^ N contents were observed in the roots and shoots of *B. pilosa* in three ^15^N_2_-fed treatments amended by these two isolates or their mixture than their control counterparts. This suggests that more bioavailable ^15^ N was produced by the diazotrophic endophytes via BNF, and the plants subsequently utilized bioavailable ^15^ N. In addition, the relative abundances of the *nifH* transcripts were higher in the shoots and roots of three treatments amended by bacteria than controls, suggesting that nitrogenase gene expression was stimulated after amending these bacteria. Therefore, the amendment of diazotrophic endophytes substantially enhanced the BNF, further promoting the growth of *B. pilosa*.

A combination of qPCR and the amplicon-based metagenomic study was performed to assess the ability of these diazotrophic endophytes to colonize internal plant tissues. qPCR and 16S rRNA-based microbial community analyses revealed significantly higher relative abundances of *Pseudomonas* and *Rhizobium* in the rhizosphere and endosphere of treatments amended by *Pseudomonas* and *Rhizobium*, respectively (Fig. 5). Successful colonization of exogenous plant-growth-promoting bacteria was reported to play a critical role in promoting plant growth [58]. Host plants likely have different mechanisms to recruit bacterial endophytes (please see “Results” section for more information [59–61]). However, there are still unknown mechanisms for the recruitment of diazotrophic endophytes by tailing pioneer plants. Such information is important because it may provide guidance to improve the colonization of diazotrophs and thus promote plant growth. Plants can release photosynthates or exudates from their roots, which can initiate early communication between plants and bacterial endophytes that consequently steers the colonization process [62–64]. Unfortunately, it was impractical to detect photosynthates or exudates in this study due to the small amount of tailing samples, most of which were used for DNA extraction and isotope analysis. Moreover, bacterial quorum sensing may contribute to colonization [59]. A recent study showed that a quorum-sensing mutant of *Burkholderia phytofirmans* PsJN could not efficiently colonize *Arabidopsis thaliana* and did not increase its growth [65]. In this study, both the *Pseudomonas* and *Rhizobium*-associated MAGs harbor the quorum-sensing system regulator gene LuxR (Fig. 3B), implying that these bacteria may have the potential for quorum sensing.

The exopolysaccharides (EPS) synthesized by bacterial cells may promote root surface attachment and colonization [66, 67]. For example, mutations in the EPS synthesis gene reduced the colonization efficiency of *Gluconacetobacter diazotrophicus*. However, colonization ability was rescued by the external addition of wild-type produced EPS [68]. Both the *Pseudomonas* and *Rhizobium* MAGs contained essential genes to produce EPS, suggesting their biofilm formation and plant colonization capabilities. Detoxification of reactive oxygen species (ROS) frequently occurs during the early stage of endophyte colonization [69]. ROS-deactivating genes, superoxide dismutase and glutathione reductase, were substantially expressed by the diazotrophic endophyte *Gluconacetobacter diazotrophicus* during the early stages of rice root colonization [70]. The current study also detected ROS-deactivating genes in MAGs associated with *Pseudomonas* and *Rhizobium*. Taking all these observations together, the potential to deactivate ROS, produce EPS, and interact through quorum sensing may contribute to plant colonization by *Pseudomonas* and *Rhizobium*. It is worth noting that the detection of these genes does not guarantee that they perform the corresponding functions in plants. Further *in planta* experiments is necessary to reveal paths and mechanisms of *B. pilosa* to recruit these bacteria.

## Conclusions

Diazotrophic endophytes can promote plant growth, particularly those growing in oligotrophic environments such as mine tailings. A combination of field microbial community characterization, DNA-SIP analysis, and pot experiments suggested that diazotrophic endophytes can promote the growth of pioneer plants in mine tailings. It is suggested that several diazotrophic endophytes, especially the bacteria of the genera *Pseudomonas* and *Rhizobium*, can promote the pioneer plants growing mine tailings. Essential genes for plant growth promotion and nitrogen fixation were found in MAGs associated with these two bacterial isolates, suggesting their metabolic potential to promote plant growth.

The pot experiment indicated that the amendment of *Rhizobium* sp. G-14 and *Pseudomonas* sp. Y-5, two diazotrophic endophytes isolated from *M. sinensis*, can promote the growth of another pioneer plant *B. pilosa* growing in mine tailings. The ^15^ N isotope analysis and quantification of *nifH* transcription demonstrated the amendment of these bacteria significantly increased the activity of N fixation in plants and subsequently increased the plant growth. Microbial community analysis indicated that these two bacteria enriched within the rhizosphere, and colonize within endosphere of *B. pilosa.* Metagenomic-binning suggested that some genes responsible for quorum sensing, EPS formation, and ROS detoxification were detected in MAGs associated with *Pseudomonas* and *Rhizobium*, suggesting that these bacteria may use these mechanisms for plant colonization. In addition to pot experiments, field-scale experiments are expected to verify the capability of diazotrophic endophytes to promote *M. sinensis* in actual mine tailings. Importantly, *M. sinensis* has been considered for bioenergy production [33, 71], this research may not only increase phytoremediation efficiency but also promote bioenergy production development on contaminated soils. In conclusion, our findings revealed that the specialized keystone taxa participate in BNF, which provides an excellent opportunity to apply these keystone taxa as microbial agents for tailings bioremediation.

## Supplementary Information

**Additional file 1: Table S1.** The chemical properties of sample sites. **Table S2.** Node table for biotic interaction network in the less contaminatedsites. **Table S3.** Edge table for biotic interaction network in the lesscontaminated sites. **Table S4.** Node table for biotic interaction network in thehigh contaminated sites. **Table S5.** Edge table for biotic interaction network inthe high contaminated sites. 

**Additional file 2:** The methods of "Extraction of root endophyticmicroorganisms", "SIP gradient fractionation", "Shotgunmetagenome sequencing", and "Growth promotion potential of isolateddiazotrophs". **Figure S1.** Overview of the experimental design employing acombination of field study, DNA-SIP, and pot experiment. **Figure S2.** Samplinglocations for pioneer plant *M. sinensis* in southern China. **Figure S3.** Theassays of checking root surface sterilization. **Figure S4.** Experimental design of*Bidens pilosa* L. plants grown in the 50 mL serum tube sealed with septa. **Figure S5.** Comparison of the distribution of the different phyla between lowcontaminated and the high contaminated sites. **Figure S6.** The relative abundanceof *nifH*/16S rRNA genes in the low contaminated and the high contaminatedsites. **Figure S7.** The isolated diazotrophic strains. **Figure S8.** The PCoA plots ofbeta similarities measured as Bray-Curtis distances for bacterial community inthe rhizosphere and root endosphere. **Figure S9.** Comparison of the relativeabundances of *Pseudomonas* and *Rhizobium* among differenttreatments in rhizosphere, root, and shoot using qPCR. 

## Data Availability

The raw data have been submitted to the NCBI database for public use (accession no PRJNA818089).
