# Correction: Characterization of diazotrophic root endophytes in Chinese silvergrass (Miscanthus sinensis)

**DOI:** 10.1186/s40168-022-01445-2

**Published:** 2022-12-16

**Authors:** Yongbin Li, Rui Yang, Max M. Häggblom, Mengyan Li, Lifang Guo, Baoqin Li, Max Kolton, Zhiguo Cao, Mohsen Soleimani, Zheng Chen, Zhimin Xu, Wenlong Gao, Bei Yan, Weimin Sun

**Affiliations:** 1 Guangdong Key Laboratory of Integrated Agro-Environmental Pollution Control and Management, Institute of Eco-Environmental and Soil Sciences, National-Regional Joint Engineering Research Center for Soil Pollution Control and Remediation in South China, Guangdong Academy of Sciences, Guangzhou, 510650 China; 2 Joint Laboratory for Environmental Pollution and Control, Guangdong-Hong Kong-Macao, Guangzhou Institute of Geochemistry, Chinese Academy of Sciences, Guangzhou, 510640 China; 3 Department of Biochemistry and Microbiology, Rutgers University, New Brunswick, NJ 08901 USA; 4 Department of Chemistry and Environmental Science, New Jersey Institute of Technology, Newark, NJ 07102 USA; 5 French Associates Institute for Agriculture and Biotechnology of Drylands, Ben-Gurion University, of the Negev, Beer Sheva, Israel; 6 School of Environment, Key Laboratory of Yellow River and Huai River Water Environment and Pollution Control, Ministry of Education, Henan Normal University, Xinxiang, 453007 China; 7 Department of Natural Resources, Isfahan University of Technology, Isfahan, Iran; 8 Department of Health and Environmental Sciences, Xi’an Jiaotong-Liverpool University, Suzhou, 215123 China; 9 Engineering and Technology Research Center for Agricultural Land Pollution Prevention and Control of Guangdong Higher Education Institutes, College of Resources and Environment, Zhongkai University of Agriculture and Engineering, Guangzhou, 510225 China

**Correction: Microbiome 10, 186 (2022)**

**https://doi.org/10.1186/s40168-022-01379-9**

Following publication of the original article [1], the authors identified an error in the author name of Mohsen Soleimani.

The incorrect author name is: Mohsen Solemani

The correct author name is: Mohsen Soleimani

The author group has been updated above and the original article [1] has been corrected.
