# Supplementary material for: Characterization of diazotrophic root endophytes in Chinese silvergrass (Miscanthus sinensis)
Source: Microbiome. 2022 Nov 3;10:186. doi: 10.1186/s40168-022-01379-9 (PMC9632085; doi:10.1186/s40168-022-01379-9)
Supplement: Supplementary file 3 — Additional file 2: The methods of "Extraction of root endophyticmicroorganisms", "SIP gradient fractionation", "Shotgunmetagenome sequencing", and "Growth promotion potential of isolateddiazotrophs". Figure S1. Overview of the experimental design employing acombination of field study, DNA-SIP, and pot experiment. Figure S2. Samplinglocations for pioneer plant M. sinensis in southern China. Figure S3. Theassays of checking root surface sterilization. Figure S4. Experimental design ofBidens pilosa L. plants grown in the 50 mL serum tube sealed with septa. Figure S5. Comparison of the distribution of the different phyla between lowcontaminated and the high contaminated sites. Figure S6. The relative abundanceof nifH/16S rRNA genes in the low contaminated and the high contaminatedsites. Figure S7. The isolated diazotrophic strains. Figure S8. The PCoA plots ofbeta similarities measured as Bray-Curtis distances for bacterial community inthe rhizosphere and root endosphere. Figure S9. Comparison of the relativeabundances of Pseudomonas and Rhizobium among differenttreatments in rhizosphere, root, and shoot using qPCR. [file 40168_2022_1379_MOESM2_ESM.docx]

*Supplementary Information (SI) for*

**Characterization of diazotrophic root endophytes in** **Chinese silvergrass (*Miscanthus sinensis)***

Yongbin Li^1,2#^, Rui Yang^1#^, Max M. Häggblom^3^, Mengyan Li^4^, Lifang Guo^1^, Baoqin Li^1^, Max Kolton^1,2,5^, Zhiguo Cao^6^, Mohsen Solemani^7^, Zheng Chen^8^, Zhimin Xu^9^, Wenlong Gao^1,2^, Bei Yan^1,2^, Weimin Sun^1,2*^

^1^ National-Regional Joint Engineering Research Center for Soil Pollution Control and Remediation in South China, Guangdong Key Laboratory of Integrated Agro-environmental Pollution Control and Management, Institute of Eco-environmental and Soil Sciences, Guangdong Academy of Sciences, Guangzhou 510650, China

^2^  Guangdong-Hong Kong-Macao Joint Laboratory for Environmental Pollution and Control, Guangzhou Institute of Geochemistry, Chinese Academy of Sciences, Guangzhou 510640, China

^3^ Department of Biochemistry and Microbiology, Rutgers University, New Brunswick, NJ 08901, USA

^4^ Department of Chemistry and Environmental Science, New Jersey Institute of Technology, Newark, NJ 07102, USA

^5^ French Associates Institute for Agriculture and Biotechnology of Drylands, Ben-Gurion University of the Negev, Beer Sheva, Israel

^6^ School of Environment, Key Laboratory of Yellow River and Huai River Water Environment and Pollution Control, Ministry of Education, Henan Normal University, Xinxiang 453007, China

^7^ Department of Natural Resources, Isfahan University of Technology, Iran

^8^ Department of Health and Environmental Sciences, Xi'an Jiaotong-Liverpool University, Suzhou 215123, China.

^9^ Engineering and Technology Research Center for Agricultural Land Pollution Prevention and Control of Guangdong Higher Education Institutes, College of Resources and Environment, Zhongkai University of Agriculture and Engineering, Guangzhou, 510225, China

#Y.L. and L.G. contributed equally to this paper.

*Corresponding authors:

Dr. Weimin Sun

Phone: 86-020-87024633

Fax: 86-020-87024123

Email: wmsun@soil.gd.cn

808 Tianyuan Road, Guangzhou, Guangdong, China

**This supporting information contains 17 pages, materials and methods, and 9 figures.**

Extraction of root endophytic microorganisms......................................................................................................S4

SIP gradient fractionation......................................................................................................................................S5

Shotgun metagenome sequencing..........................................................................................................................S5

Growth promotion potential of isolated diazotrophs..............................................................................................S6

Fig. S1. Overview of the experimental design employing a combination of field study, DNA-SIP, and pot experiment.............................................................................................................................................................S8

Fig. S2. Sampling locations for pioneer plant *M. sinensis* in southern China.........................................................S9

Fig. S3. The assays of checking root surface sterilization.....................................................................................S10

Fig. S4. Experimental design of *M. sinensis* plants grown in the 50 mL serum tube sealed with septa. The plants were then collected for ^15^N isotope analysis.........................................................................................................S11

Fig. S5. Comparison of the distribution of the different phyla between low contaminated (LC) and the high contaminated (HC) sites.......................................................................................................................................S12

Fig. S6 The relative abundance of *nifH*/16S rRNA genes in the low contaminated (LC) and the high contaminated (HC) sites.............................................................................................................................................................S13

Fig. S7. The isolated diazotrophic strains.............................................................................................................S14

Fig. S8. The PCoA plots of beta similarities measured as Bray-Curtis distances for bacterial community in the rhizosphere (A) and root endosphere (B)..............................................................................................................S15

Fig. S9. Comparison of the relative abundances of Pseudomonas (A, B, C) and Rhizobium (D, E, F) among different treatments in rhizosphere, root, and shoot using qPCR .........................................................................S16

References............................................................................................................................................................S17

**Methods**

***Extraction of root endophytic microorganisms***

The process of removing the root surface microorganisms: 1) Root samples were washed 5 times with sterile buffer (10 mM MgSO_4_, hereafter) for 5 min each on a MIX-200 Multi-tube Vortexer (TUOHE, Shanghai, China) (The last wash buffer was retained and labeled as R1); 2) Roots were ultrasonically washed once with buffer (adding 0.01% (v:v) Tween 20) for 15 min on an SB-5200D ultrasonic cleaner (SCIENTZ, Zhejiang, China), then the roots were washed twice with sterile buffer for 5 min each on a Multi-tube Vortexer (The last wash buffer was retained and labeled as R2); 3) Roots were washed once with 1% NaClO solution (adding 0.01% (v:v) Tween 20) for 20 min on a Multi-tube Vortexer, and then were ultrasonically washed once for 15 min. Finally, the roots were washed 5 times with sterile buffer for 5 min each on a Multi-tube Vortexer (The last wash buffer was retained and labeled as R3). The surface sterilization was checked by applying 1 mL of wash buffer (R1, R2, R3) to LB agar plates, which were incubated at 28°C for 7 days.

The extraction root endophytes: 1) The surface-sterilized root samples were homogenized in the sterile buffer using a sterile blender, and were subjected to filter using sterile Miracloth (EMD Millipore) to remove large plant tissue; 3) The filtrate was centrifuged at 500 × *g* for 10 min at 10°C to collect the supernatant, which was centrifuged at 9000 × *g* for 15 min at 10°C; 4) The supernatant was discarded, and the pellet was resuspended in 25 mL sterile saline, carefully layered over 10 ml of Nycodenz solution (Axis-Shield, Oslo, Norway; density:1.3 g mL^-1^) in sterile centrifuge tubes, and then centrifuged at 15,000 × *g* for 60 min at 10°C; 5) Approximately 1 ml containing microbial cells was carefully pipetted from the band at the interface of buffer and Nycodenz, transferred to a sterile tube and mixed with an equal volume of sterile saline, and then centrifuged at 7,500 × g for 20 min. The supernatant was discarded, and this centrifugation step was repeated once. Finally, the pellet was resuspended in 5 mL sterile saline to gain the endophytic microorganisms for DNA-SIP inoculation.

***SIP gradient fractionation***

Specifically, approximately 4,000 ng gDNA was mixed with CsCl gradients in 4.9 mL OptiSeal polyallomer tubes (Beckman Coulter, Palo Alto, USA) to yield an initial buoyant density (BD) of 1.714 g mL^-1^. The mixture was centrifuged at 408,500*g* for 48h at 20°C in an Optima XPN-100 Ultracentrifuge equipped with a VTi 90 vertical rotor (Beckman Coulter, USA). The obtained DNA gradients were fractionated into 24 equal volumes (200 uL per fraction) using a fraction recovery system (Beckman Coulter, USA). The BD of each fraction was obtained by measuring the refractive index using a digital refractometer (Palette, ATAGO, Japan). Nucleic acids were precipitated from CsCl gradients with glycogen (6 μL) (ZOMANBIO, China) in 30% ethanol and then eluted with 30 μL of TE buffer (pH 8.0). The relative proportion of putative diazotrophs in every fraction to the total 24 fractions was assessed using the abundance of the *nifH* gene. Three representative DNA samples of the heavy or light fractions containing the highest *nifH* gene abundance from the duplicate culture of the SIP incubation treatments were pooled as composites, respectively.

***Shotgun metagenome sequencing***

A total of 10.5 Gb raw reads generated were qualified using Trimmomatic [1]. A total of 85,796,444 (12.5 Gb) qualified reads were then *de novo* assembled using Megahit with a range of k-mer values from 21 to 121 at the step of 10 [2]. Binning of the assembled metagenome was computed and refined using MaxBIN2 and metaBAT2 in MetaWRAP [3]. Completion and contamination of all the bins were estimated by CheckM [4]. The metagenome-assembled genomes (MAGs) with completion > 70% and contamination < 10% were subjected to taxonomy annotation using MiGA [5] and genes annotation using KofamKOALA against the KEGG database with default parameters [6], respectively. In addition, 23 reference genomes of *Serratia* were obtained from the NCBI database (Table S3), and functional genes were annotated using KofamKOALA. The phylogenetic trees, including *Serratia*-associated MAGs and *Serratia* reference genomes, were analyzed. Functional genes involved in As(III) oxidation and N_2_ fixation were identified based on protein sequences using CVTree3 (<http://cvtree.online/v4/prok/index.html>) [7].

***Growth promotion potential of isolated diazotrophs***

A pot experiment was employed to assess the growth promotion potential and colonization ability of the isolated diazotrophs on *B*. *pilosa*. Seeds of *B. pilosa* were collected from an uncontaminated site located in Guangdong (23°18′69′′N, 113°36′24′E). The plants were planted in the mixture of tailings, which were collected from a stibnite mine from XKS, and vermiculite (v:v = 1:1). In addition, the isolated diazotrophic strains were inoculated in LB broth and cultured at 30°C for 36 h at 180 rpm, respectively. The culture was centrifuged at 6,000 rpm for 5 min. Then the cell pellets were washed 3 times with sterile normal saline and adjusted to 10^8^ cells mL^-1^ with sterile normal saline.

The seeds of *B. pilosa* were surface sterilized with NaClO (10 % v/v) for 10 min, followed by rinsing with sterile deionized water. One hundred seedlings were sown in each plastic pot. *Rhizobium* sp. G-14 and *Pseudomonas* sp. Y-5 were isolated and identified as diazotrophic endophytes. Therefore, these bacteria were inoculated to *B. pilosa*. Four different treatments in triplicate were established as follows: (i) inoculation with *Rhizobium* sp. G-14 (designated as Rhi.Inoc.), (ii) inoculation with *Pseudomonas* sp. Y-5 (designated as Pseu. Inoc.), (iii) inoculation with the mixture of *Rhizobium* sp. G-14 and *Pseudomonas* sp. Y-5 (designated as Mix. Inoc.), and (iv) treatments without inoculating pure isolates (designated as control). It is worth noting that all inoculated bacteria are native to the tested site as they were isolated and enriched from plant samples from the same site. Thirty-milliliter bacterial suspension (or deionized water for control) was applied to the pots. On day 7, 30 ml of the bacterial suspension (or deionized water) was re-applied to the pot. Pots were placed in a greenhouse under humidity and temperature control. The seedlings were watered (50 mL deionized water in each pot) every 5 days until harvest. No fertilizer was applied. At day 35, plant samples, including rhizosphere soil, root, and shoot, were harvested from each treatment and total plant N and length were recorded.

Samples from different compartments (rhizosphere soil, roots, and shoots) were collected on days 7, 14, 21, and 28 to analyze the ability of the inoculant bacteria to colonize plants and express the *nifH* gene. Total genomic DNAs were extracted from different compartments on days 7, 14, 21, and 28 as described previously [8]. The bacterial community in the rhizosphere and root endosphere were analyzed by Illumina MiSeq sequencing of 16S rRNA gene as described above. In addition, the relative abundances of *Rhizobium* and *Pseudomonas* were quantified by qPCR with the primers as described previously [8]. In addition, in order to assess the expression of the *nifH* gene, total RNA was extracted from plant tissues using RNAiso Plus reagent (TaKaRa, Kyoto, Japan). Subsequently, RNA was digested with DNase I and reversely transcribed into cDNA using PrimeScript^TM^ RT reagent kit (RaKaRa, Kyoto, Japan). The *nifH* primer and method for RT-qPCR were described previously [9].


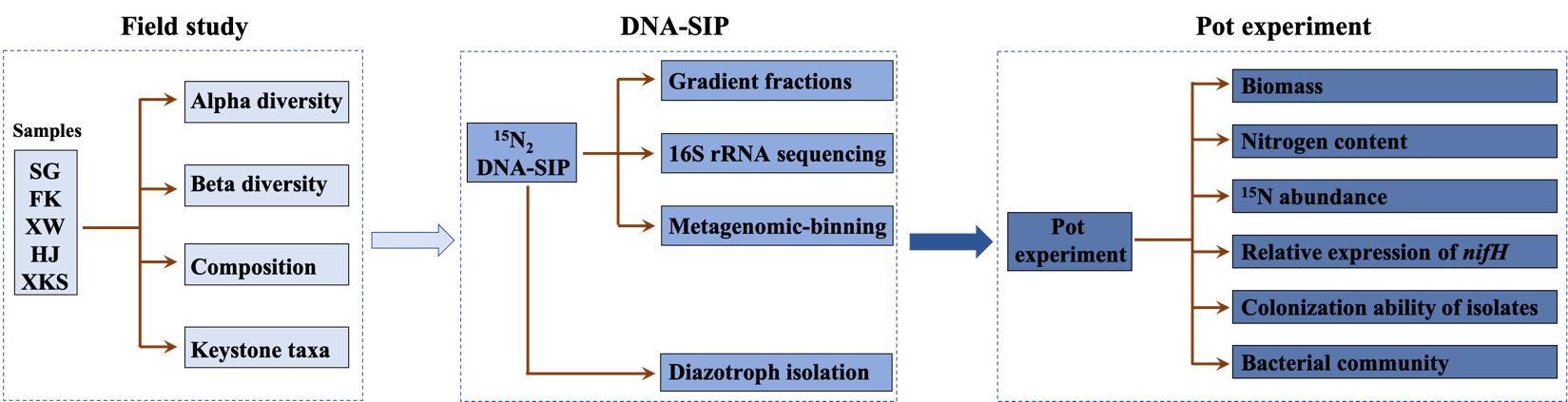


**Fig. S1.** Overview of the experimental design employing a combination of field study, DNA-SIP, and pot experiment. SG: Shaoguang, FK: Fankou, XW: Xiuwen, HJ: Huangjia, XKS:Xikuangshan.


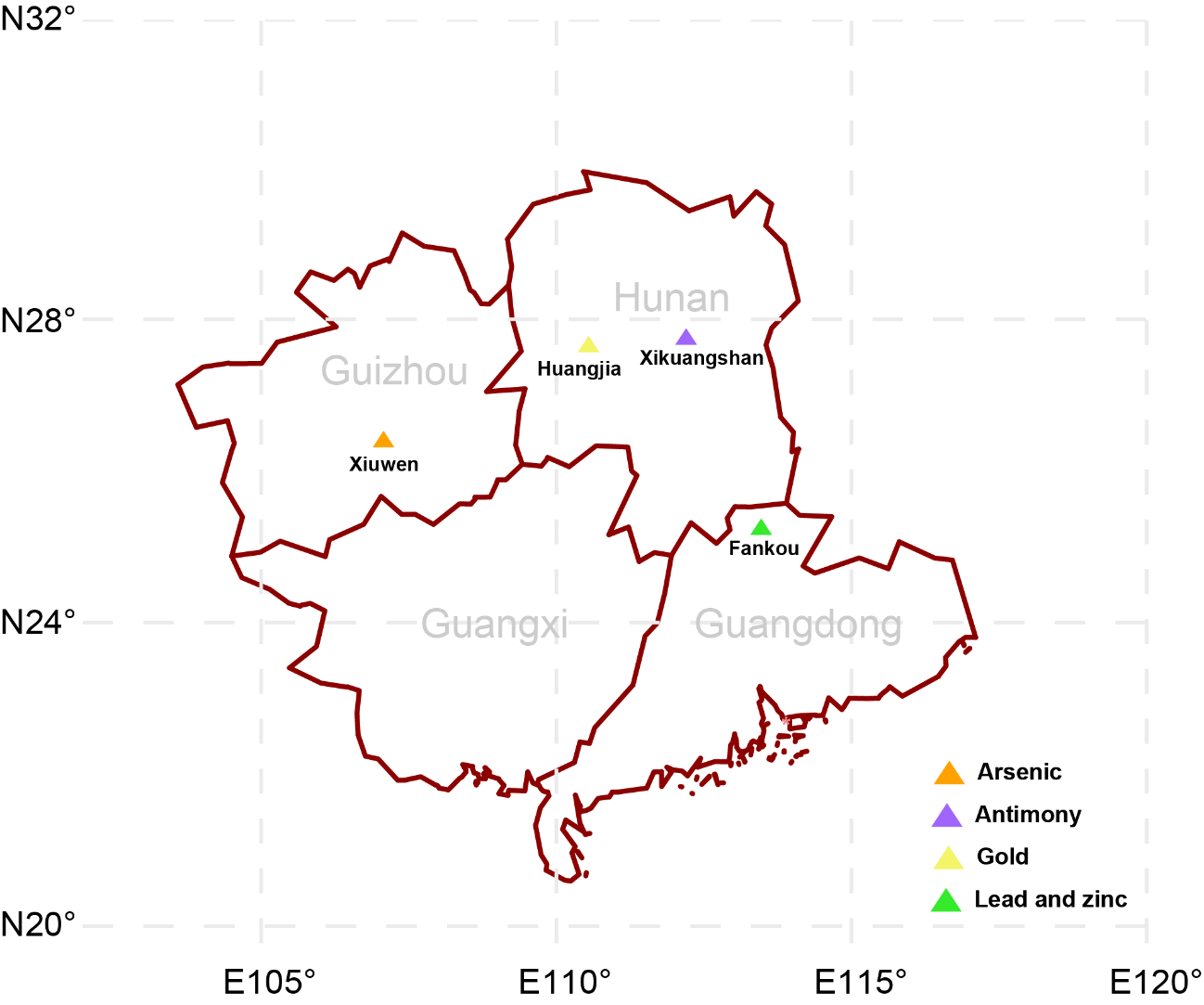


**Fig. S2.** Sampling locations for pioneer plant *M. sinensis* in southern China.


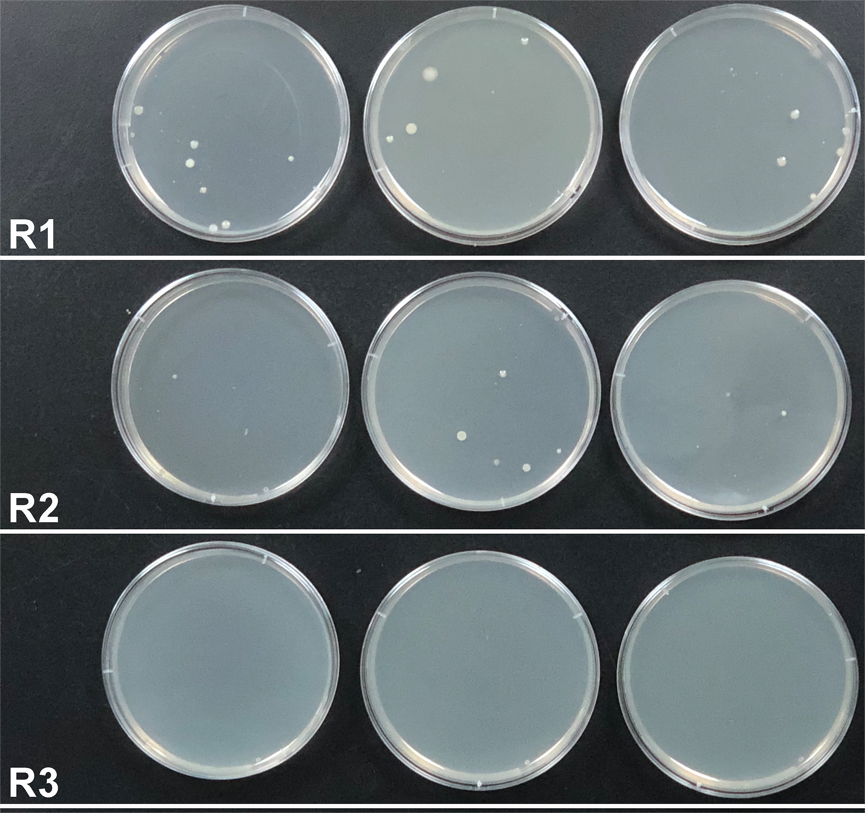


**Fig. S3.** The assays of checking root surface sterilization.


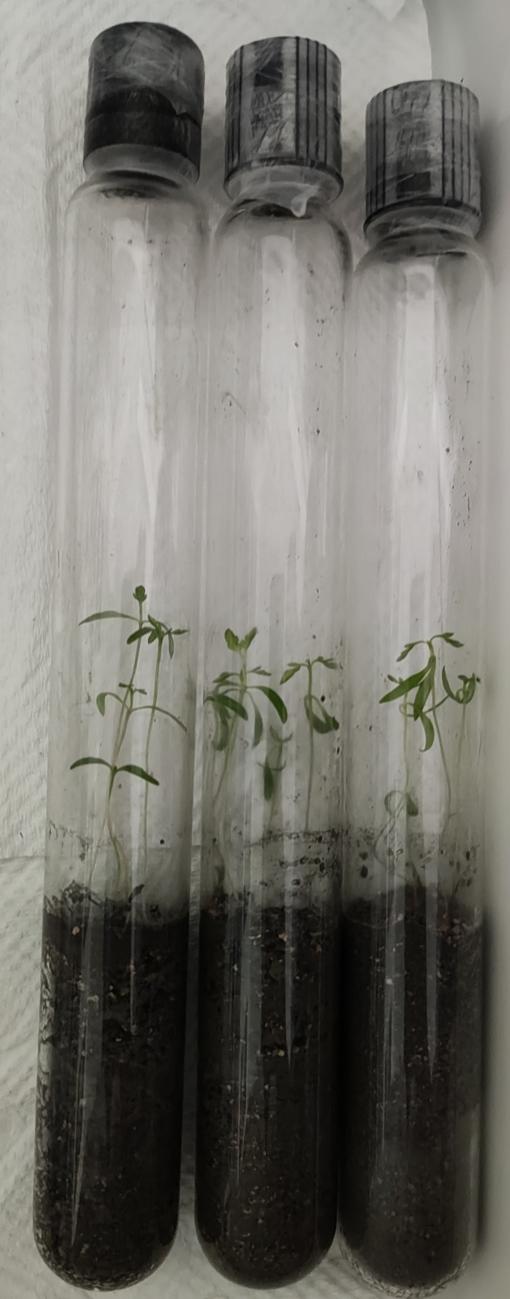


**Fig. S4.** Experimental design of *Bidens pilosa* L. plants grown in the 50 mL serum tube sealed with septa. The plants were then collected for ^15^N isotope analysis.


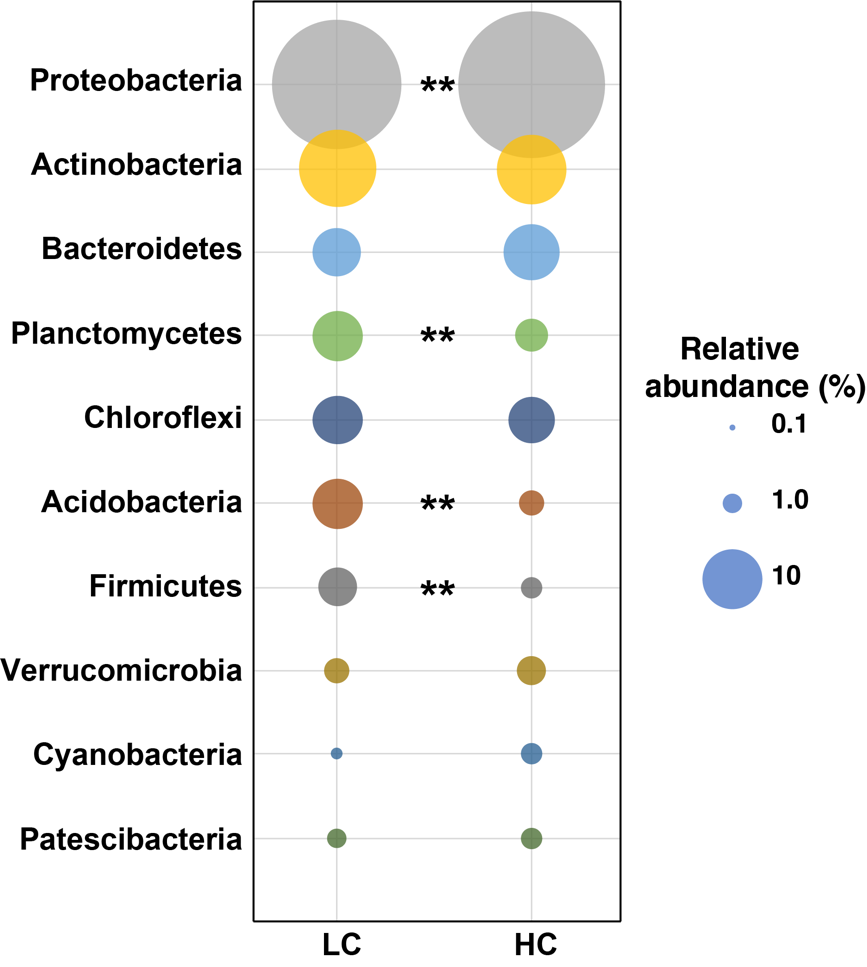


**Fig. S5.** Comparison of the distribution of the different phyla between low contaminated (LC) and the high contaminated (HC) sites. * and ** indicate significant differences between the LC and HC at *p* < 0.05 and *p* < 0.01, respectively. The bacterial community was analyzed based on 5 replicate root samples from each sampling site (a total of 20 root samples).


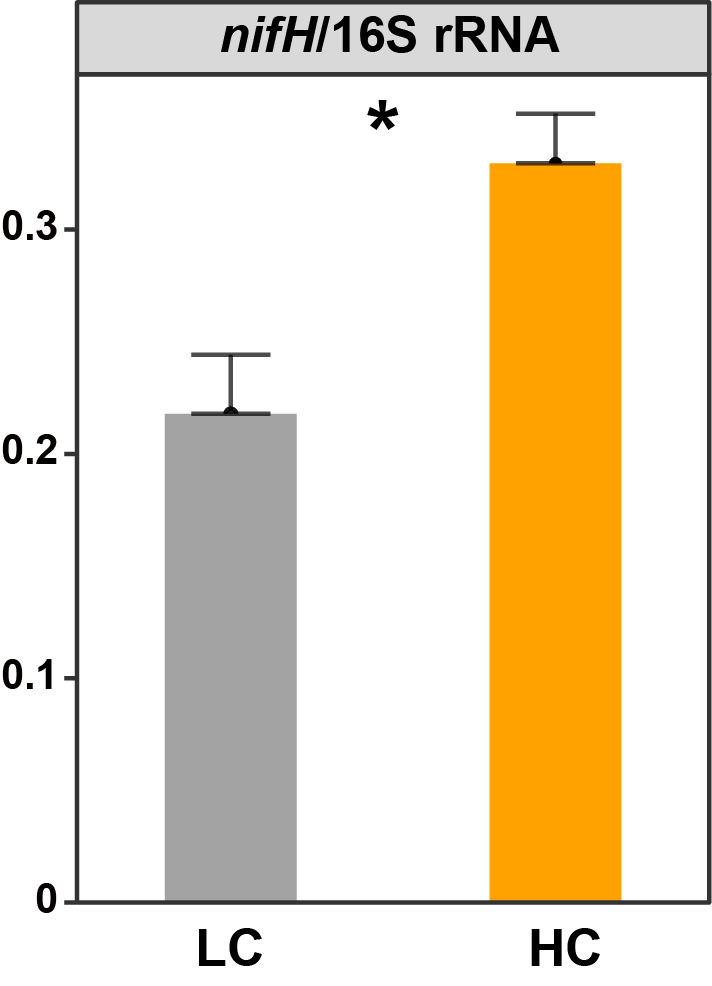


**Fig. S6** The relative abundance of *nifH*/16S rRNA genes in the low contaminated (LC) and the high contaminated (HC) sites. * indicates significant differences between the LC and HC at *p* < 0.05. Values are given as mean of 5 independent biological replicates, and the bars represent standard error.


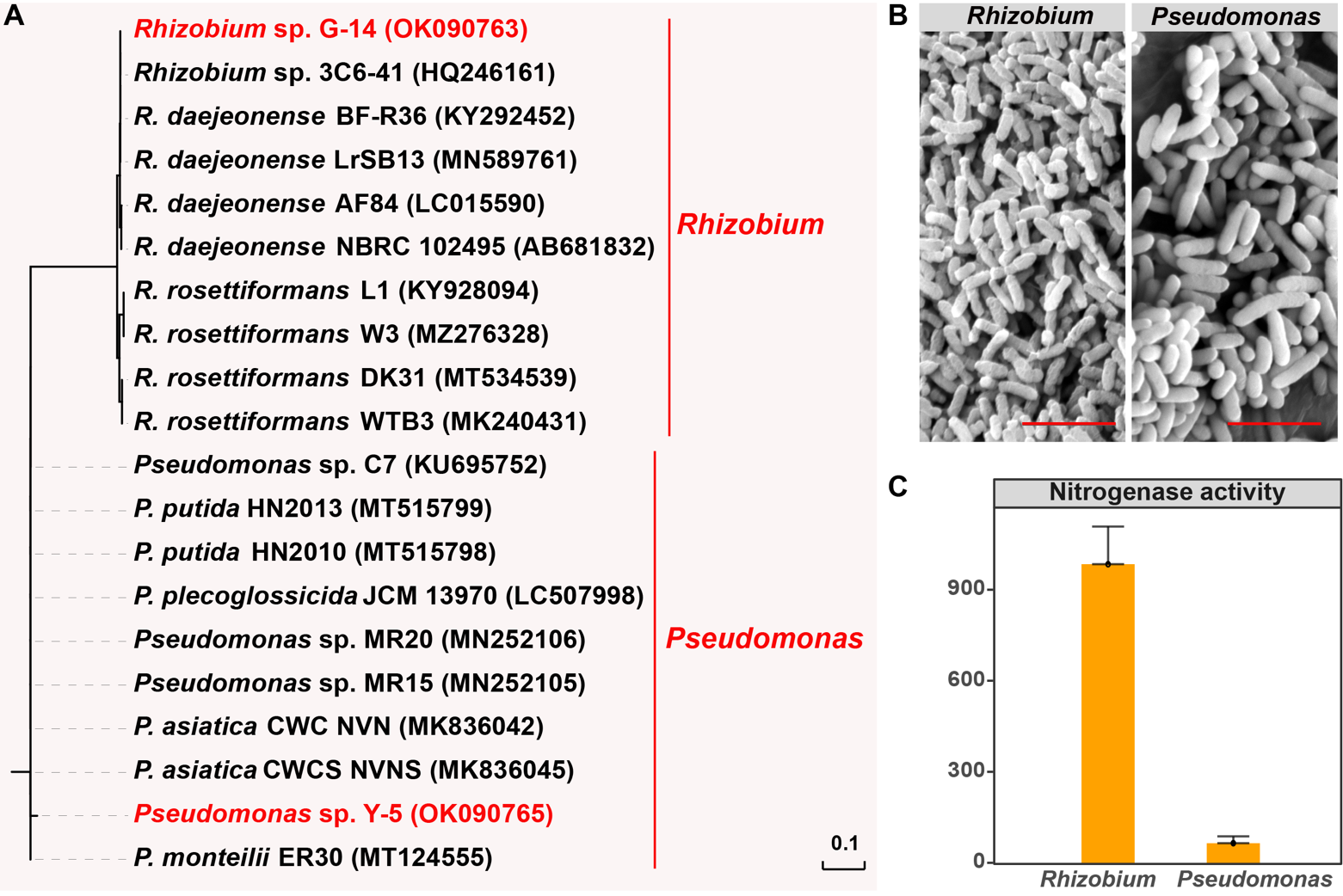


**Fig. S7.** The isolated diazotrophic strains. (A) A phylogenetic tree based on 16S rRNA sequence shows the position of isolated strains with other closely related isolates. The tree was structured using the neighbor-joining method, with the bootstrap analyses of 1,000 cycles. Strains isolated in this study are underlined with red bolded letters. (B) Scanning electron micrograph of isolated diazotrophic strains. The scale bar indicates 3 μm. (C) The nitrogenase activity of isolated strains. The unit of nitrogenase activity is nmol C_2_H_4_ mg^-1^ protein h^-1^. Values are given as a mean of 3 independent biological replicates, and the bars represent standard error.


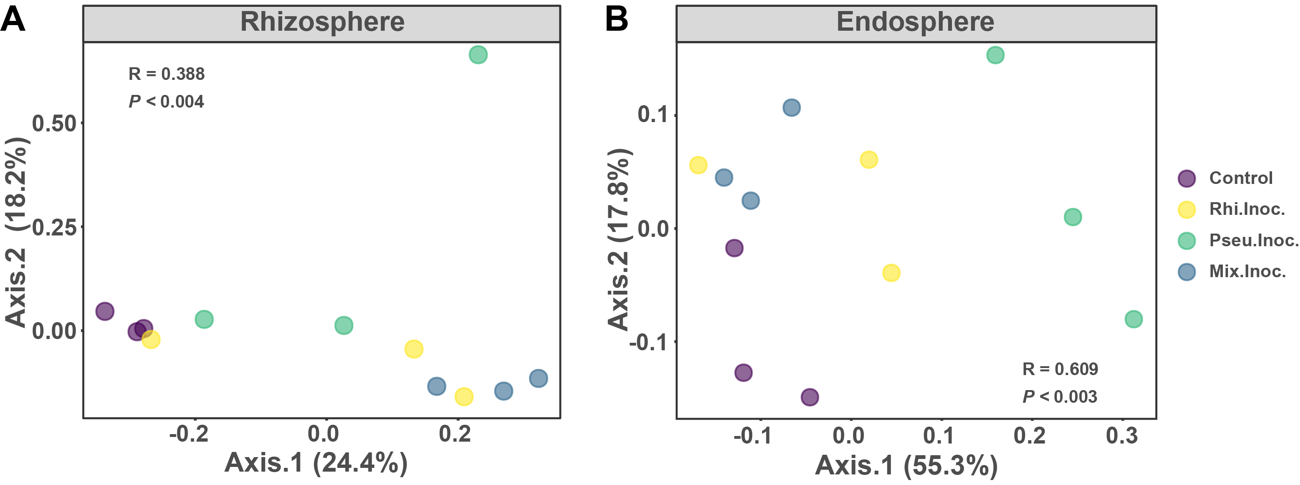


**Fig. S8.** The PCoA plots of beta diversity similarities based on Bray-Curtis distances for bacterial communities in the rhizosphere (A) and root endosphere (B). The bacterial community was analyzed based on 3 replicate samples from each treatment (a total of 24 samples).


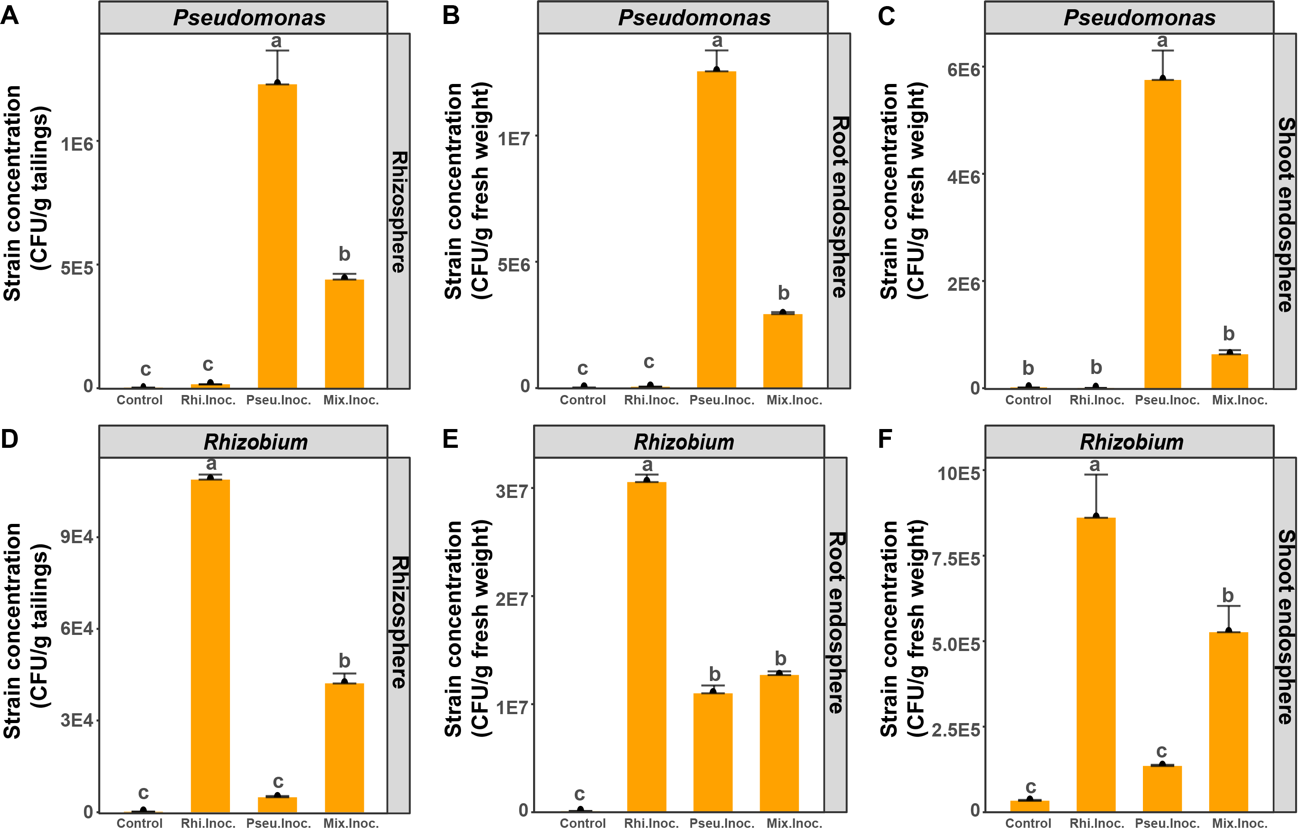


**Fig. S9.** Comparison of the relative abundances of *Pseudomonas* (A, B, C) and *Rhizobium* (D, E, F) among treatments in the rhizosphere, root, and shoot using qPCR. Values are given as a mean of 3 independent biological replicates, and the bars represent standard error. ANOVA with an LSD test (*p* < 0.05) indicates statistically significant differences denoted by different letters for each assessed parameter.

References

1. Bolger AM, Lohse M, Usadel B. Trimmomatic: a flexible trimmer for Illumina sequence data. Bioinformatics. 2014;30(15):2114-2120.

2. Li D, Liu C-M, Luo R, Sadakane K, Lam T-W. MEGAHIT: an ultra-fast single-node solution for large and complex metagenomics assembly via succinct de Bruijn graph. Bioinformatics. 2015;31(10):1674-1676.

3. Gherman U, Jocelyne D, James T. MetaWRAP-a flexible pipeline for genome-resolved metagenomic data analysis. Microbiome. 2018; 6(1): 1-13

4. Parks DH, Imelfort M, Skennerton CT, Hugenholtz P, Tyson GW. CheckM: assessing the quality of microbial genomes recovered from isolates, single cells, and metagenomes. Genome Res. 2015;25(7):1043-1055.

5. Rodriguez RL, Gunturu S, Harvey WT, Rosselló-Mora R, Tiedje JM, Cole JR, et al. The Microbial Genomes Atlas (MiGA) webserver: taxonomic and gene diversity analysis of Archaea and Bacteria at the whole genome level. Nucleic Acids Res. 2018;46(W1):W282-W288.

6. Aramaki T, Blanc-Mathieu R, Endo H, Ohkubo K, Kanehisa M, Goto S, et al. KofamKOALA: KEGG Ortholog assignment based on profile HMM and adaptive score threshold. Bioinformatics. 2020;36(7):2251-2252.

7. Zuo G, Hao B. CVTree3 web server for whole-genome-based and alignment-free prokaryotic phylogeny and taxonomy. Genom Proteom Bioinf. 2015;13(5):321-331.

8. Li Y, Li Y, Zhang H, Wang M, Chen S. Diazotrophic *paenibacillus beijingensis* BJ-18 brovides nitrogen for plant and promotes plant growth, nitrogen uptake and metabolism. Front Microbiol. 2019;10:1119

9. Yongbin L, Qin L, Guohua G, Sanfeng C. Phosphate solubilizing bacteria stimulate wheat rhizosphere and endosphere biological nitrogen fixation by improving phosphorus content. PeerJ. 2020;8:e9062.
